# Zinc-induced Self-association of Complement C3b and Factor H: IMPLICATIONS FOR INFLAMMATION AND AGE-RELATED MACULAR DEGENERATION*

**DOI:** 10.1074/jbc.M113.476143

**Published:** 2013-05-09

**Authors:** Ruodan Nan, Stuart Tetchner, Elizabeth Rodriguez, Po-Jung Pao, Jayesh Gor, Imre Lengyel, Stephen J. Perkins

**Affiliations:** From the ‡ Department of Structural and Molecular Biology, Darwin Building, University College London, Gower Street, London WC1E 6BT, United Kingdom and; the § Department of Ocular Biology and Therapeutics, UCL Institute of Ophthalmology, 11-43 Bath Street, London EC1V 9EL, United Kingdom

**Keywords:** Analytical Ultracentrifugation, Complement, Inflammation, X-ray Scattering, Zinc, Age-related Macular Degeneration

## Abstract

The sub-retinal pigment epithelial deposits that are a hallmark of age-related macular degeneration contain both C3b and millimolar levels of zinc. C3 is the central protein of complement, whereas C3u is formed by the spontaneous hydrolysis of the thioester bridge in C3. During activation, C3 is cleaved to form active C3b, then C3b is inactivated by Factor I and Factor H to form the C3c and C3d fragments. The interaction of zinc with C3 was quantified using analytical ultracentrifugation and x-ray scattering. C3, C3u, and C3b associated strongly in >100 μm zinc, whereas C3c and C3d showed weak association. With zinc, C3 forms soluble oligomers, whereas C3u and C3b precipitate. We conclude that the C3, C3u, and C3b association with zinc depended on the relative positions of C3d and C3c in each protein. Computational predictions showed that putative weak zinc binding sites with different capacities exist in all five proteins, in agreement with experiments. Factor H forms large oligomers in >10 μm zinc. In contrast to C3b or Factor H alone, the solubility of the central C3b-Factor H complex was much reduced at 60 μm zinc and even more so at >100 μm zinc. The removal of the C3b-Factor H complex by zinc explains the reduced C3u/C3b inactivation rates by zinc. Zinc-induced precipitation may contribute to the initial development of sub-retinal pigment epithelial deposits in the retina as well as reducing the progression to advanced age-related macular degeneration in higher risk patients.

## Teaser

**Background:** Sub-retinal pigment epithelial deposits contain complement proteins and bioavailable zinc.

**Results:** Ultracentrifugation and x-ray scattering show that >100 μm zinc induces oligomer formation in each of C3, C3u, and C3b, in analogy to Factor H.

**Conclusion:** Factor H-C3b complexes are precipitated by zinc, which inhibits complement activation.

**Significance:** A potential molecular mechanism for zinc-induced sub-retinal deposit formation is clarified.

## Introduction

**FIGURE 1.**
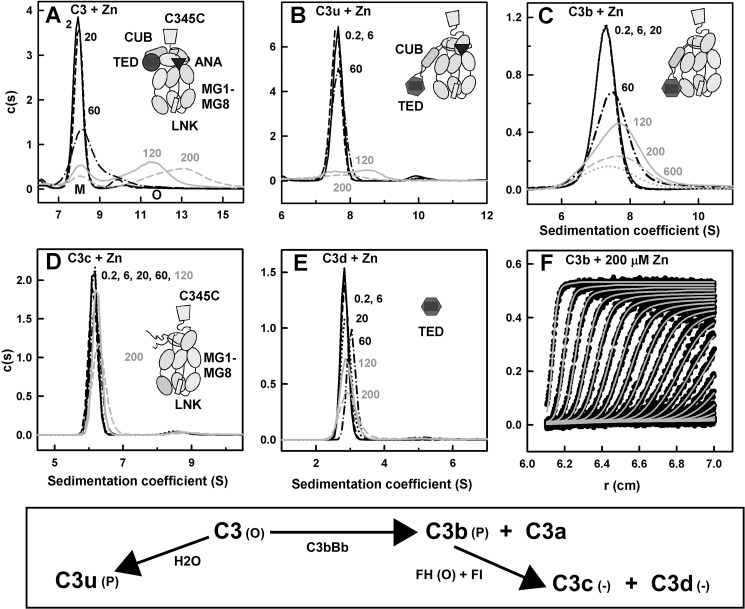
**Sedimentation velocity analyses of C3, C3u, C3b, C3c, and C3d in the presence of zinc.** C3, C3u, C3b, and C3c were studied at 4.4 μm, and C3d was studied at 7.8 μm. The 10–13 domains of C3, C3u, C3b, and C3c are depicted as schematics with the TED (*circle* or *hexagon*) and ANA (*triangle*) domains shown in *dark outlines* when present. Beneath the *c*(*s*) plots, a schematic summary of the relationships between the five forms of C3 is shown. Oligomers, precipitates, or no changes upon adding zinc are denoted by *P*, *O*, and −, respectively. *A*, the *c*(*s*) sedimentation coefficient distribution analysis for C3 with zinc concentrations at 2 μm (*solid black line*), 20 μm (*black dashes*), 60 μm (*black dots and dashes*), 120 μm (*solid gray line*), and 200 μm (*gray dashes*). The monomer peak is denoted as *M*, and the oligomer peaks at high *S* values are denoted as *O*. Here and below, the zinc concentrations in μm are denoted numerically. *B*, the *c*(*s*) analysis for C3u with zinc concentrations at 0.2 μm (*line*) and 6 μm (*dashes*) and three higher zinc concentrations are shown as in *A. C*, the *c*(*s*) analysis for C3b, with zinc concentrations at 0.2 μm (*line*), 6 μm (*dashes*), and 20 μm (*dots*), and three higher zinc concentrations are shown as in *A. D*, the *c*(*s*) analysis for C3c with zinc concentrations are shown as in *C. E*, the *c*(*s*) analysis for C3d with zinc concentrations are shown as in *C. F*, shown are the sedimentation boundary fits for C3b with 200 μm zinc; all 30 scans are shown. The experimental absorbance data are shown as *black circles*, whereas the boundary fits are shown as gray lines.

Activation of the complement system through the classical, lectin and alternative pathways leads to the conversion of C3^3^ to C3b (1, 2). C3 is the most abundant complement protein in plasma (about 1.0 mg/ml or 5.3 μm) and is an acute phase protein whose concentration increases during inflammation. C3 is a member of the α2-macroglobulin family (3). Unactivated C3 consists of 13 domains, namely 8 macroglobulin domains MG1 to MG8, a linker domain (LNK), an anaphylatoxin domain (ANA; C3a), a complement C1r/C1s-UEGF-BMP1 domain (CUB), a thioester-containing domain (TED; C3d), and a C345C domain (Fig. 1*A*) (4). The spontaneous hydrolysis of the thioester bond in the TED/C3d domain leads to C3u (also known as C3_H2_O or C3i), also with 13 domains (Fig. 1*B*). The formation of convertase enzyme complexes (such as those originating from complement Factor B) cleaves the ANA/C3a domain from C3 to form C3b. Conformational changes occur in C3b to expose the highly reactive thioester bond, which enables C3b to bind covalently to the cell surface (3). Despite structural similarities with C3b, C3u is unable to bind to surfaces due to its hydrolyzed thioester bond. When C3b is bound to the pathogen cell surface, a positive-feedback amplification leading to increased C3b production occurs (2). After C3b binding to Factor H (FH), C3b is cleaved by Factor I to yield inactive C3c and C3dg, hence regulating the amounts of active C3b (1, 2). FH consists of 20 short complement regulator (SCR) domains. The N-terminal domains SCR-1/4 bind to C3b between its TED/CUB and MG domains (5), whereas the C-terminal domains SCR-19/20 bind to the TED/C3d domain of C3b (6, 7). Additional C3b binding sites may occur in the middle of FH (8, 9).

The complement proteins including C3 and FH are genetically associated with age-related macular degeneration (AMD) (10–19). AMD is a leading cause of visual impairment in the elderly in the western world (11, 13). A hallmark of AMD is the appearance of sub-retinal pigment epithelial deposits (sRPEds) within Bruch's membrane, an extracellular matrix layer between the retinal pigment epithelium (RPE) and the choroidal microvasculature (20–22). sRPEds contain oxidized lipids, carbohydrates, and >100 proteins, including the complement components and trace elements (11, 23, 24). Zinc is the second most abundant trace mineral in the human body, and ocular tissues contain unusually high concentrations of zinc (25, 26). The secretion of zinc from zinc-rich RPE cells is elevated under oxidative stress (27). High millimolar levels of zinc were identified in sRPEds and Bruch's membrane using x-ray fluorescence, with some in a bio-available form as shown by fluorescent probes (28). Interestingly, zinc inhibits the cleavage of C3b by FH, and FH aggregates in the presence of zinc (29, 30). Recently, both heterozygous and homozygous FH were each shown to self-associate indefinitely through its SCR-6/8 domains with a dissociation constant of ∼10 μm (31, 32). Although zinc has no measurable effect on Factor I, zinc was reported to bind to C3 (33, 34). Here, to complete our studies of the FH-zinc complexes, we have now quantified the comparative effects of zinc on the self-association of C3, C3u, C3b, C3c, and C3d and its effect on the centrally important regulatory C3b-FH complex.

Zinc binding sites at protein interfaces are often formed from His, Asp, Glu, and/or Cys residues (35). A shared zinc site between two protein surfaces involves between one to three residues from each surface. To determine the extent to which surface-bound zinc causes each of C3, C3u, C3b, C3c, and C3d to self-associate, we employed analytical ultracentrifugation and synchrotron x-ray scattering (36) combined with metal binding-site predictions for these proteins (37). In comparison to our recent FH-zinc studies (31, 32), we found here that the C3, C3u, and C3b oligomerize with a zinc concentration of >100 μm in a 10-fold weaker manner than the zinc-induced oligomerization of FH. For the key regulatory C3b-FH complex, we showed that zinc leads to the precipitation of this complex at >100 μm zinc concentrations. Our results explain why C3b cleavage by FH and Factor I is inhibited by zinc (31, 34). Molecular mechanisms are suggested for the initial formation of sRPEds that lead to AMD as well as an explanation for the role of zinc in reducing the occurrence of developing advanced AMD in high risk patients (38, 39).

## EXPERIMENTAL PROCEDURES

### 

#### 

##### Protein Purification and Concentrations

Wild-type C3 was purified from fresh human plasma by anion-exchange using a Q-Sepharose fast-flow column (Amersham Biosciences) and a Mono Q 5/50 GL column (GE Healthcare) (40). C3u was produced by incubating C3 with 200 mm hydrazine for 2 h at 37 °C in a water bath and leaving this overnight at 4 °C. C3b was produced by treating 1 mg/ml C3 in HEPES buffer (10 mm HEPES, 137 mm NaCl, 0.5 mm EDTA, pH 7.4) with 10 μg/ml trypsin (1% w/w enzyme/substrate) for 120 s at 37 °C in a water bath, then adding 40 μg/ml soybean trypsin inhibitor to stop cleavage before transferring onto ice. To block the free SH group of the C3b thioester, 20 mm iodoacetamide was added to the mixture, then this was incubated in the dark at 20 °C for 30 min (5). The C3b sample was diluted in Tris buffer (25 mm Tris, 140 mm NaCl, 0.5 mm EDTA, pH 8.0), then concentrated immediately and passed through a Superose^TM^ 6 prep grade XK 16/60 size-exclusion column. C3c was prepared by incubating outdated human plasma for 7 days at 37 °C in a water bath, then following the same protocol for the purification of C3 to produce C3c. C3u and C3b (but not C3) were active in functional assays using Factor I and Factor H (31). Recombinant C3d with a GST tag was expressed in *Escherichia coli* and purified using a GSTrap FF 5-ml column (GE Healthcare) connected to a HiTrap Benzamidine FF (high sub) 1-ml column (GE Healthcare) (41). Wild-type FH was purified from outdated human plasma using monoclonal affinity chromatography (32). The absorbance coefficients for C3, C3u, C3b, C3c, C3d, and FH (1%, 280 nm, 1 cm path length) were calculated from their compositions to be 9.40, 9.40, 9.83, 9.21, 13.15, and 16.2, respectively, assuming the presence of three high mannose type oligosaccharides at Asn-63, Asn-917, and Asn-1597 in C3 (42, 43). Molecular masses were calculated from compositions to be 189.0 kDa for C3 and C3u, 179.3 kDa for C3b, 135.7 kDa for C3c, 34.6 kDa for C3d, and 154.4 kDa for FH. All proteins were passed through a size-exclusion gel filtration column to remove potential aggregates immediately before the addition of zinc, then dialyzed into HEPES buffer without EDTA. Each protein was routinely checked by SDS-PAGE before and after the ultracentrifugation and scattering experiments. Complement hemolytic activity assays were performed in triplicate using an alternative pathway kit based on the lysis of chicken erythrocytes in an agarose gel (Binding Site Group Ltd., Birmingham, UK). The diameter of the zones of lysis was measured using a jewelers' eyepiece as a measure of complement activation.

##### Sedimentation Velocity Data Collection and Analyses

Analytical ultracentrifugation data were obtained on two Beckman XL-I instruments equipped with AnTi50 rotors using two-sector cells with column heights of 12 mm at a rotor speed of 50,000 rpm. Sedimentation velocity experiments at 20 °C were performed with C3 at 0.76 mg/ml (4.0 μm), C3u at 0.87 mg/ml (4.6 μm), C3b at 0.79 mg/ml (4.4 μm), C3c at 0.6 mg/ml (4.4 μm), and C3d at 0.27 mg/ml (7.8 μm). Zinc titrations utilized ZnSO_4_ at concentrations of 0.2, 6, 20, 60, 120, 200, and 600 μm. The C3b-FH complex was formed by incubating C3b at 0.99 mg/ml (5.5 μm) with FH at 0.85 mg/ml (5.5 μm) at 4 °C overnight followed by sedimentation velocity the following morning at a rotor speed of 50,000 rpm at 20 °C. Interference data were analyzed for C3, C3u, and the C3b-FH complex titrated with zinc, and absorbance data were analyzed for C3b, C3c, and C3d titrated with zinc using SEDFIT software (Version 14.1) (44, 45). The size distribution analyses *c*(*s*) provided size and shape data for each species present by directly fitting the observed sedimentation boundaries to the Lamm equation using 300 interference boundaries for C3, C3u, and C3b-FH and 25–80 absorbance boundaries for C3b, C3c, and C3d. The *c*(*s*) analyses were based on a fixed resolution of 200 in which the meniscus, the bottom of the cell, the base line, and the average frictional ratio *f*/*fo* were floated until the overall root mean square deviation and the fits between the observed and calculated sedimentation boundaries were satisfactory. The starting *f*/*fo* values were between 1.35 to 1.4 for C3, C3u, C3b, and C3c, 1.2 for C3d, and 1.78 for the C3b-FH complex, Monomers and oligomers of C3, C3u, C3b, C3c, and C3d were quantitated using the integration function in the *c*(*s*) analyses. The integrations assumed that the signal intensities of the monomer at the lowest zinc concentrations is 100%. Other details are described elsewhere (31, 46).

##### X-ray Scattering Data Collection and Analyses

X-ray scattering data were acquired in two beam sessions on Instrument ID02 at the European Synchrotron Radiation Facility (Grenoble, France) operating with a ring energy of 6.0 gigaelectron volts in 4-bunch and 16-bunch mode to reduce the incident flux (47). The sample-detector distance was 3.0 m, and the x-ray wavelength was 0.0995 nm. Potential radiation damage was eliminated by the continuous movement of the sample in a flow cell during beam exposure, the use of 10 time frames of duration between 0.1 s and 0.5 s each during each acquisition, and on-line checks for the absence of radiation damage at low *Q*. In the first beam session, C3 and C3u were at 0.76 mg/ml (4 μm), C3b was at 0.72 mg/ml (4 μm), C3c was at 0.54 mg/ml (4 μm), and C3d was at 0.50 mg/ml (14.5 μm), all in HEPES buffer. In the second beam session, C3b was at 0.90 mg/ml (5.0 μm), FH was at 0.77 mg/ml (5.0 μm), and the C3b-FH complex was formed by incubating the mixture at 4 °C 2 h before adding zinc, all in HEPES buffer with 0.5 mm Pefabloc-SC^70^. ZnSO_4_ was added at concentrations of 2, 6, 20, 60, 120, 200, and 600 μm up to 2 h before the measurements. Other details including data reduction are described elsewhere (46, 48).

In a given solute-solvent contrast, the radius of gyration *R_G_* corresponds to the mean square distance of scattering elements from their center of gravity and is a measure of structural elongation. Guinier analyses at low *Q* values gives the *R_G_* value and the forward scattering at zero angle *I*(*0*) from the expression (49), 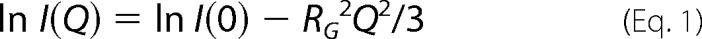 This expression is valid in a *Q.R_G_* range up to 1.5. The *I*(*0*)/*c* value (c is the protein concentration in mg/ml) is proportional to the relative molecular mass *M*_r_. The Guinier analyses were performed using an interactive PERL script program SCTPL7 (J. T. Eaton and S. J. Perkins, unpublished software) on Silicon Graphics OCTANE Workstations.

Indirect transformation of the *I*(*Q*) curve measured in reciprocal space into real space gives the distance distribution function *P*(*r*) and was carried out using the program GNOM (50), 
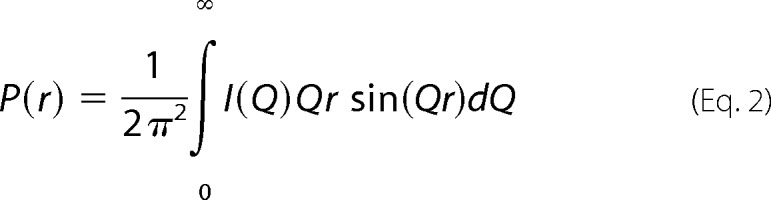

*P*(*r*) corresponds to the distribution of distances *r* between volume elements and gives an alternative calculation of the *R_G_* and *I*(*0*) values based on the full scattering curve *I*(*Q*). It also gives the most frequently occurring distance *M* within the macromolecule and the maximum dimension of the macromolecule *L*. For C3, C3u, C3b, C3c, and C3d titrated with zinc, the x-ray curves utilized up to 320 data points in the *Q* range between 0.09 and 1.50 nm^−1^. Other details are described elsewhere (31, 46, 48).

##### Prediction of Zinc Binding Sites

Potential zinc binding sites were predicted utilizing the METSITE server (37). METSITE predicts binding sites for different metals utilizing a broad set of structural identifiers. For a given metal, the characterization of the secondary structure, solvent accessibility, position-specific scoring matrix, and distance matrix ascertains those residues most likely to interact strongly with metal. METSITE utilizes the distances between the Cβ atoms of amino acid residues except for Gly residues when Cα atoms were used. For this project, the capacity of METSITE was expanded by Dr. Daniel Buchan and Prof. David T. Jones to process larger protein structures. The predictions used crystal structures for C3 (PDB code 2A73) (4), C3b (PDB codes 2I07 and 2ICF) (51, 52), C3c (PDB code 2A74) (4), and C3d (PDB codes 1C3D and 1GHQ (53, 54), and a solution structure for C3u (PDB code 3MMQ) (43). In METSITE, the false positive rate was set to 5%, and the predicted metal was set to zinc. For structures with multiple chains, all chains were relabeled to be chain A to circumvent a METSITE limitation of only being able to process single chain structures. His, Glu, Asp, and Cys residues with neural network scores greater than 0.7 were accepted as potential zinc binding residues, whereas Arg and Gly residues were removed because they do not bind zinc (35). The METSITE output was summarized in tabular form in which a neural network residue score of 0.7 represents a log likelihood ratio of ∼1. At a log likelihood ratio of 2, there are 100 correct predictions for every false positive, indicating a high level of confidence (37).

## RESULTS

### 

#### 

##### Sedimentation Velocity of C3, C3u, C3b, C3c, and C3d with Zinc

C3, C3u, C3b, and C3c were studied at concentrations of 4.0–4.6 μm, which are comparable with the physiological C3 concentration of about 1.0 mg/ml (5.3 μm) in plasma (2). C3d was studied at 0.27 mg/ml (7.8 μm), this being the lowest concentration that produced analyzable data. HEPES buffer was used to avoid the precipitation of zinc that occurs if phosphate buffer is used. Each protein was titrated using a concentration range of 0.2–600 μm ZnSO_4_.

Analytical ultracentrifugation studies the sedimentation behavior of macromolecules when they are subjected to a high centrifugal force to determine their sizes and shapes (55). This method is advantageous for the detection of multiple species that are present. In sedimentation velocity experiments with C3, C3u, C3b, C3c, and C3d, each was titrated with zinc. Good fits to the sedimentation boundaries in all cases (Fig. 1*F*) resulted in well defined size distribution analyses *c*(*s*). The five proteins each showed different sedimentation behavior as the zinc concentrations increased.

For C3 titrated with zinc, the monomer peak was consistently observed at an *s*_20,_*_w_* value of 8.42 ± 0.10 S with a molecular mass of 182 ± 4 kDa at all zinc concentrations (Fig. 1*A*). Both values agree well with those of C3 without zinc at 8.49 ± 0.03 S and 192 ± 8 kDa and with the sequence-determined molecular mass of 189.0 kDa (43).^4^ The intensity of the C3 monomer peak decreased as the zinc concentrations increased. This indicated the presence of unperturbed C3 monomer even with a 40-fold excess of zinc, meaning the binding of zinc is weak. Starting from a zinc concentration of 60 μm, the monomer peak broadened, and a second broad peak appeared that corresponded to soluble C3 oligomers with higher *s*_20,_*_w_* values of 10.2 S at 60 μm zinc, 12.3 S at 120 μm zinc, and 13.7 S at 200 μm zinc (Fig. 1*A*). The apparent molecular masses of these peaks were 244, 321, and 378 kDa, respectively, suggesting that progressive C3 oligomer formation as dimer or trimer or higher has occurred in the presence of excess zinc.

For C3u titrated with zinc, the monomer peak was consistently observed at an *s*_20,_*_w_* value of 8.05 ± 0.11 S with a molecular mass value of 176 ± 3 kDa at all zinc concentrations. These values agreed with those of C3u without zinc at 8.08 ± 0.03 S and 172 ± 16 kDa (43). Unlike C3, the C3u monomer peak almost disappeared as the zinc concentration increased to 120 and 200 μm, and no significant oligomer peaks were observed at high zinc concentrations (Fig. 1*B*). This showed that C3u precipitated in the presence of excess zinc.

For C3b, the monomer peak was observed at a *s*_20,_*_w_* value of 7.41 ± 0.01 S and a molecular mass value of 160 ± 0.3 kDa for zinc concentration to 20 μm (Fig. 1*C*). At zinc concentrations of 60 μm and above, the monomer *s*_20,_*_w_* value increased slightly, from 7.62 S at 60 μm zinc to 7.76 S at 200 μm zinc. The peak shift is attributed to the onset of C3b oligomer formation in the presence of zinc, this being less pronounced than that observed for C3. At the same time, the C3b peak intensity also decreased as the zinc concentrations increased, indicating precipitation. No separate oligomer peak was observed for C3b-zinc (Fig. 1*C*). Thus C3b behaved differently from C3 or C3u in the presence of zinc.

For C3c, the monomer peak was observed at a *s*_20,_*_w_* value of 6.51 ± 0.07 S with a molecular mass value of 121 ± 2 kDa at all zinc concentrations. This agrees with that for C3c in the absence of zinc. No change in *s*_20,_*_w_* value or intensity was observed until zinc concentrations reached 200 μm when the monomer peak became broader, this being attributed to the onset of C3c oligomer formation (Fig. 1*D*).

For C3d, the monomer was consistently observed at a *s*_20,_*_w_* value of 3.13 ± 0.09 S with a molecular mass of 34 ± 1 kDa, and no significant oligomer peak was visible. These data agree well with the *s*_20,_*_w_* value of 3.0 ± 0.1 S and a molecular mass value of 34 ± 4 kDa for monomeric C3d in 137 mm NaCl (56). Starting at a zinc concentration of 60 μm, the C3d monomer peak decreased in intensity as the zinc concentration increased but less so than C3, C3u, and C3b (Fig. 1*E*).

**FIGURE 2.**
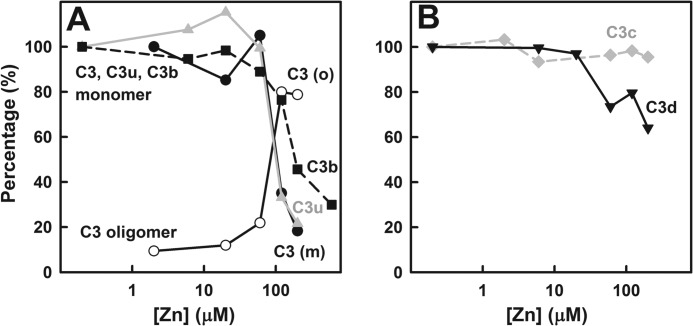
**The percentages of the monomers and oligomers of the five C3 proteins as a function of zinc concentrations.** These were determined from the *c*(*s*) analyses of Fig. 1. *A*, C3 (*filled circle*, monomer; *open circle*, oligomer, *black line*); C3u (*gray triangle*, monomer; *gray line*); C3b (*filled square*, monomer, *black dashes*). *B*, C3c (*gray diamond*, monomer; *gray dashes*); C3d (*black inverted triangle*, monomer, *black line*).

The integration of the monomer peak intensities in the *c*(*s*) analyses permitted comparison of the effect of zinc on the solubility of the five proteins. For reason of clarity, the integrations were normalized to 100% at 0–2 μm zinc. C3, C3u, and C3b decreased significantly at zinc concentrations of 120 μm and above with similar apparent protein-zinc dissociation constants *K_D_* of around 100 μm (Fig. 2*A*). C3 and C3u decreased more strongly in intensity than C3b at zinc concentrations of 120 μm and above. Of those three proteins, only C3 formed soluble oligomers that increased to ∼80% of the C3 present. This increase in C3 oligomer matched the decrease of the C3 monomer for zinc concentrations of 120 μm and above. Unlike C3, C3u, and C3b, the C3c monomer was not affected in intensity by the increase in zinc concentrations up to 200 μm, whereas the amount of the C3d monomer was slightly reduced at zinc concentrations above 60 μm, and 64% of C3d remained as a soluble monomer in 200 μm zinc (Fig. 2*B*). Neither C3c nor C3d precipitated or formed large oligomers in the manner seen for C3, C3u, and C3b. It was concluded that the presence of both the C3d and C3c regions within C3, C3u, and C3b is required for these to self-associate with zinc even though either C3d or C3c on their own do not self-associate with zinc.

To assess whether other plasma proteins undergo self-association in the presence of zinc, 0.32 mg/ml (4 μm) human serum albumin in HEPES buffer was titrated with seven concentrations of ZnSO_4_ between 0.2 and 600 μm. The *c*(*s*) analyses revealed one major peak at a *s*_20,_*_w_* value of 4.95 ± 0.06 S and a molecular mass value of 67 ± 1 kDa that was unaltered in 0.2–600 μm zinc (data not shown). This indicated that human serum albumin does not self-associate in the presence of zinc.

##### Sedimentation Velocity of the C3b-FH Complex with Zinc

**FIGURE 3.**
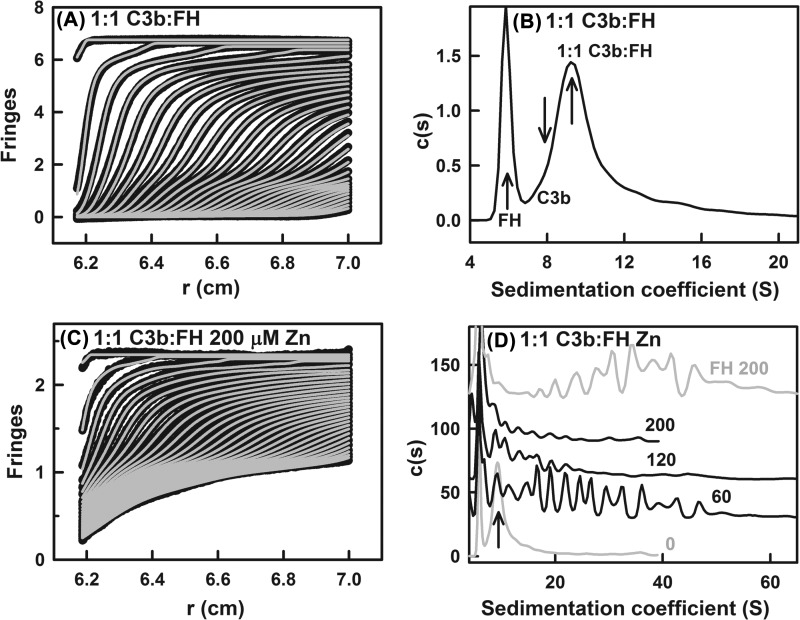
**Sedimentation velocity analyses of the C3b-FH complex in the presence of zinc.** *A*, shown are the boundary fits for the 1:1 mixture of 5.5 μm C3b and 5.5 μm FH. Only every tenth scan of the 300 scans is shown for clarity. The absorbance data are shown as *black circles*, whereas boundary fits are shown as *gray lines. B*, shown is the *c*(*s*) sedimentation coefficient distribution analysis for the 1:1 mixture of C3b and FH. The peak positions for the FH monomer (5.77 S), C3b monomer (8.16 S), and the 1:1 C3b-FH complex (9.55 S) are arrowed. *C*, the boundary fits for the 1:1 mixture of 5.5 μm FH-C3b and 5.5 μm FH with 200 μm zinc following the representation of *A. D*, the *c*(*s*) analyses for the 1:1 mixture of FH:C3b with 60 μm, 120 μm, and 200 μm zinc (*black outlines*) are shown. The analyses are normalized relative to the FH monomer peak. The zinc concentrations are denoted numerically. The peak position of the 1:1 FH-C3b complex is shown with an arrow. For comparison, the two *c*(*s*) curves (*gray outlines*) for the complex from *B* with no zinc present and for FH alone with 200 μm zinc from Fig. 4*D* of (31) are shown to the same scale.

Sedimentation velocity experiments were performed on a 1:1 mixture of FH at 0.85 mg/ml (5.5 μm) and C3b at 0.99 mg/ml (5.5 μm) (Fig. 3*A*). The *K_D_* value is 0.6–1.6 μm for the FH-C3b complex (9). Accordingly, 60–70% FH-C3b complex formation is expected. Unbound monomeric FH was observed at a *s*_20,_*_w_* value of 5.58 S, and this corresponded to a molecular mass value of 143 kDa, which is close to the sequence-predicted mass of 154 kDa (Fig. 3*B*). The peak with a *s*_20,_*_w_* value of 9.18 S and a molecular mass value of 287 kDa was attributed to the 1:1 complex of FH-C3b with a sequence-predicted molecular mass value of 334 kDa. Unbound monomeric C3b was an unresolved shoulder at a *s*_20,_*_w_* value of ∼7.5 S that overlapped with the major peak at 9.18 S. Small amounts of other species were observed at *s*_20,_*_w_* values higher than 10 S; however, their peaks were not well resolved and were not considered further here.

The effect of zinc on the 1:1 complex of C3b and FH was now investigated. Given that the *K_D_* for the FH-zinc interaction is ∼10 μm (31, 32) and that for C3b-zinc is ∼100 μm (Fig. 2*A*), both zinc interactions are weaker than that for the FH-C3b complex. Good fits to the sedimentation boundaries with 60, 120, and 200 μm of zinc were obtained in all cases (Fig. 3*C*). On adding zinc, the peak for monomeric FH at 5.58 S was consistently observed at all zinc concentrations (Fig. 3*D*). The peak intensity of monomeric FH decreased from 100% with no zinc to 43% at 200 μm zinc. At 60 μm zinc, multiple peaks with *s*_20,_*_w_* values up to 50 S were observed. The intensity of these oligomer peaks were much higher than for only FH with 60 μm zinc, and both the FH monomer and C3b-FH peaks decreased in intensity, indicating that these peaks correspond to a mixture of FH-Zn and C3b-FH-Zn oligomers (see Fig. 1 of Ref. 32). At 120 and 200 μm zinc, these multiple peaks showed clear decreases in intensity, showing that the C3b-FH-zinc oligomers precipitated when zinc was above 100 μm. The corresponding experiment with FH in 200 μm zinc showed that FH-zinc oligomers were much more soluble in the absence of C3b (32). In addition, the major peak for the C3b-FH complex at 9.18 S was reduced in 60 and 120 μm zinc and disappeared in 200 μm zinc. These decreased peak intensities with 120–200 μm zinc is attributed to the formation of very large C3b-FH-zinc complexes that precipitate and sediment rapidly to the bottom of the ultracentrifuge cell even before the first scan was recorded.

##### X-ray Scattering of C3, C3u, C3b, C3c, and C3d with Zinc

Small-angle x-ray scattering is a diffraction method for the study of solution structures of macromolecules in random orientations (57). The effect of zinc on freshly purified C3, C3u, C3b, C3c, and C3d were investigated by x-ray scattering. C3, C3u, C3b, and C3c were again studied at 4 μm, whereas C3d was studied at 14.5 μm (0.50 mg/ml), this being the lowest concentration of C3d that produced analyzable x-ray data. Each protein was titrated with 2–600 μm ZnSO_4_. The scattering data *I*(*Q*) showed excellent signal-noise ratios and no detectable effect from radiation damage.

The Guinier fits at low *Q* values (where *Q* = 4π sin θ/λ; 2θ = scattering angle; λ = wavelength) detect aggregates more readily than ultracentrifugation (31). The Guinier radius of gyration (*R_G_*) monitors the degree of elongation of the protein, and Guinier *I*(*0*)/*c* value is proportional to the relative molecular mass (49, 57). The fits for the five proteins were performed in a restricted *Q* range from 0.14 to 0.22 nm^−1^, even though good Guinier linearity continued to 0.45 nm^−1^ at low zinc concentrations, because this reduced *Q* range provided a sensitive monitor for aggregation.

**FIGURE 4.**
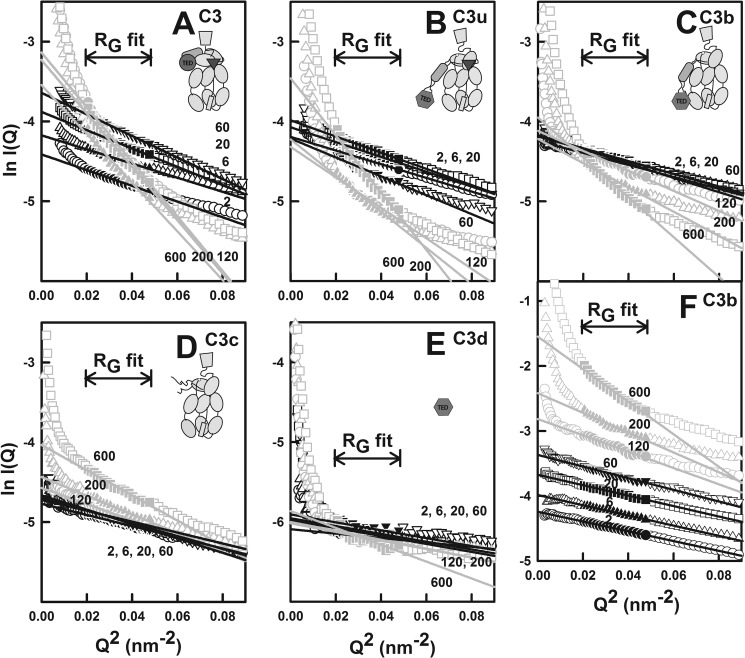
**X-ray Guinier analyses of ln *I*(*Q*) *versus Q*^2^ for C3, C3u, C3b, C3c, and C3d titrated with zinc.** For clarity, the 10–13 domains of C3, C3u, C3b, and C3c are depicted as schematics. In all panels, the *open symbols* correspond to the experimental data, and the *filled symbols* correspond to those used for the Guinier straight line fits. The *Q* fit range was 0.14–0.22 nm^−1^ in all cases (*arrow ranges*), and the zinc concentrations in micromolar are numerically labeled as shown. The Guinier *R_G_* plots are shown for C3 (*A*), C3u (*B*), C3b (*C* and *F*), and C3c (*D*), all at ∼4 μm, and for C3d (*E*) at 14.5 μm. The ZnSO_4_ concentrations were 2 μm (○), 6 μm (▵), 20 μm (□), and 60 μm (▿), all shown in *black*, and 120 μm (○), 200 μm (▵), and 600 μm (□), all shown in *gray*. In *F*, the C3b Guinier plots from *C* are displaced in steps of 0.2 log units to show the processes of aggregation and precipitation more clearly.

**FIGURE 5.**
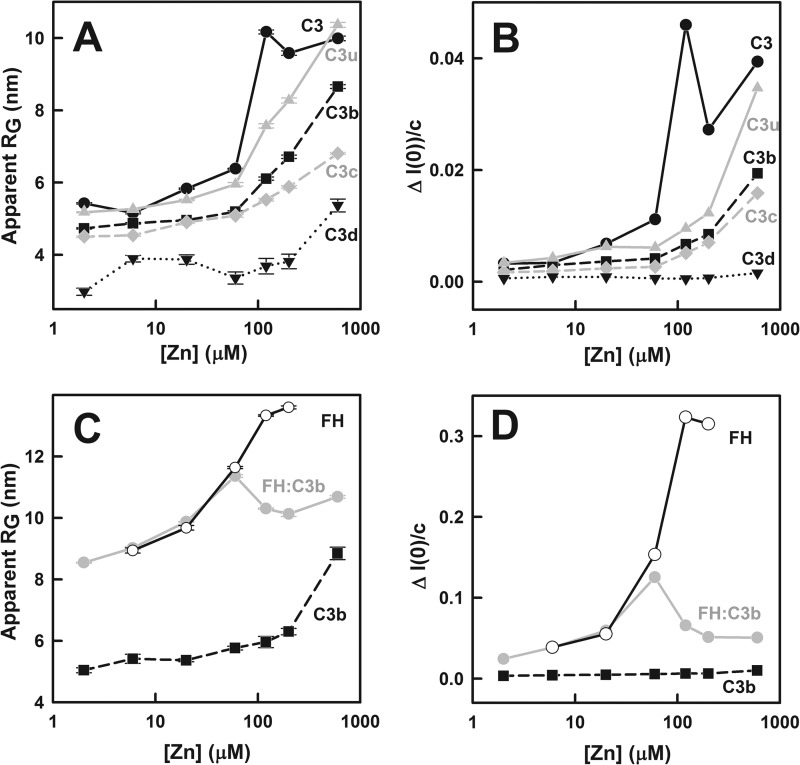
**Dependence of the Guinier *R_G_* and *I*(*0*)/*c* parameters on zinc concentrations from 2 to 600 μm.** Each value was measured in quadruplicate and averaged, and statistical *error bars* are shown where visible. *A*, the apparent *R_G_* values for C3 (*black circles*, *black line*), C3u (*gray triangles*, *gray line*), C3b (*black squares*, *black dashes*), C3c (*gray diamonds*, *gray dashes*) and C3d (*inverted black triangles*, *dotted line*) were determined from the fits of Fig. 4, *A–E. B*, shown is the change in the *I*(*0*)/*c* values between the Guinier plots, the difference based on the *Q* fit ranges of 0.14–0.22 and 0.32–0.45 nm^−1^ for C3, C3u, C3b, C3c, and C3d using the same symbols as in *A. C*, the apparent *R_G_* values for FH (*open circles*, *black line*), the FH-C3b complex (*gray circles*, *gray line*), and C3b (*black squares*, *dashed line*) measured in another beam session are compared with each other (the Guinier fits are not shown). *D*, shown is the change in the *I*(*0*)/*c* values between the Guinier plots, the difference based on the *Q* fit ranges of 0.14–0.22 and 0.32–0.45 nm^−1^ for FH, the C3b-FH complex, and C3b, using the same symbols as in *C*.

For C3 at the lowest zinc concentration of 2 μm and for 6, 20, and 60 μm zinc, the mean *R_G_* value was 5.43 ± 0.02 nm, which is higher than that of 4.52 ± 0.08 nm for native C3 without zinc in the larger *Q* range from 0.13 to 0.30 nm^−1^ (43). This increase is attributed to minor aggregation of C3, the latter being visible as a slight upturn in the scattering curve at the lowest *Q* values (Fig. 4*A*). For 120, 200, and 600 μm zinc, the *I*(*Q*) intensities increased significantly at low *Q* values as the result of zinc-induced aggregation. At larger *Q* values, these *I*(*Q*) intensities dropped for reasons of protein losses (precipitation). These *I*(*Q*) changes led to significant increases in the apparent *R_G_* value, which represents the average *R_G_* values of the monomeric and aggregated species (Fig. 5*A*) (58).

For C3u at a zinc concentration of 2 μm, the mean *R_G_* value was 5.18 ± 0.16 nm, which is close to the value of 4.88 ± 0.23 nm in the *Q* range from 0.13 to 0.30 nm^−1^ for C3u without zinc (43). Some minor aggregation was visible at the lowest *Q* values (Fig. 4*B*). As for C3, little changes occurred at 6–60 μm zinc, whereas significant increases in intensity and the apparent *R_G_* value were seen at 120–600 μm zinc (Fig. 5*A*). In this case, the larger decrease in intensities at the larger *Q* values showed that more protein precipitation of C3u occurred at 120–600 μm zinc.

For C3b with 2 μm zinc, the mean *R_G_* value was 4.73 ± 0.16 nm, which is close to that of 4.88 ± 0.23 nm above for C3u without zinc (43). As for C3 and C3u, little changes occurred for zinc concentrations at 6–60 μm. Significant increases in intensity and apparent *R_G_* value were seen for zinc concentrations at 120–600 μm together with protein precipitation seen at larger *Q* (Figs. 4*C* and 5*A*). The increased aggregation with increase in zinc concentration is more clearly seen in the curves that were replotted with offsets (Fig. 4*F*).

For C3c with 2 μm zinc, the mean *R_G_* value was 4.50 ± 0.13 nm with 2 μm zinc; this *R_G_* value is similar to those for C3u and C3b above. Unlike C3, C3u, and C3b, the scattering curves showed little change for zinc concentration up to 60 μm and only modest increases in intensities for zinc from 120 to 600 μm (Fig. 4*D*). The apparent *R_G_* increases were much smaller than those seen for C3, C3u, and C3b (Fig. 5*A*).

For C3d with 2 μm zinc, the apparent *R_G_* value of C3d was 2.98 ± 0.30 nm in the *Q* range of 0.14 to 0.22 nm^−1^ (Fig. 4*E*). C3d is small, and a larger Guinier *Q* fit range is normally used. The *R_G_* value was 1.95 nm in the *Q* fit range of 0.16–0.55 nm^−1^, in good accord with the *R_G_* value of 2.02 nm calculated from its crystal structure (59). Here, the Guinier fits with a *Q* range of 0.14- 0.22 nm^−1^ were almost unchanged between 2 and 600 μm zinc. At the lowest *Q* values below 0.14 nm^−1^, intensity increases were seen for 200 and 600 μm zinc, showing that slight aggregates have formed together with some minor precipitation seen at large *Q* (Fig. 4*E*). The apparent *R_G_* increases were the smallest compared with the other four proteins (Fig. 5*A*). It was concluded that C3d remained mostly monomeric in solution for zinc concentrations between 2 and 600 μm.

When aggregates are present, the observed scattering curve is the sum of the scattering curves of the macromolecular species present (58). The apparent *R_G_* values showed that C3 exhibited the strongest zinc-induced oligomerization followed by C3u, C3b, C3c, and C3d in that order (Fig. 5*A*). The corresponding changes in the *I*(*0*)/*c* values were best analyzed using base-line Guinier *I*(*0*)/*c* values from a second *Q* fit range between 0.32 and 0.45 nm^−1^. The difference Δ between the two *I*(*0*)/*c* values compensated for protein precipitation that reduced the *I*(*0*)/*c* values (Fig. 5*B*). The Δ*I*(*0*)/*c* values showed that C3 exhibited the strongest oligomerization followed by C3u, C3b, C3c, and C3d in that order. These are consistent with the integrations from ultracentrifugation (Fig. 2).

The distance distribution function *P*(*r*) reports on the protein shapes of the zinc-induced aggregates of the five proteins. The *P*(*r*) curve gives the distances between all pairs of atoms within the macromolecule. This leads to the most frequently occurring distance *M* from the position of the peak maximum, the maximum length *L* from the point at which *P(r*) becomes zero at large *Q*, and an independent calculation of the *R_G_* and *I*(*0*) values for comparison with the above Guinier values (57). In summary, the five *P*(*r*) analyses showed that C3, C3u, and C3b aggregated strongly with zinc concentrations of 120 μm and above, whereas C3c and C3d showed little significant effects.

**FIGURE 6.**
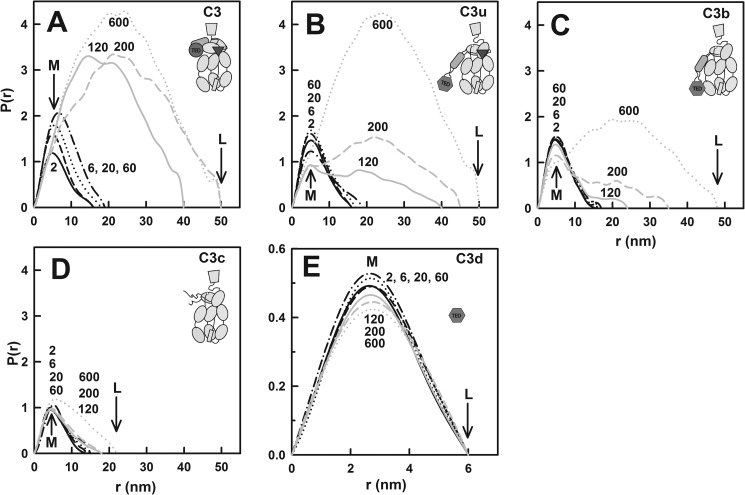
**Dependence of the distance distribution function *P(r*) of C3, C3u, C3b, C3c, and C3d on zinc concentrations from 2 to 600 μm.** For clarity, the 10–13 domains of C3, C3u, C3b, and C3c are depicted as schematics. The *P(r*) curves were calculated from the *I*(*Q*) curves used in Fig. 4. *A*, C3; *B*, C3u; *C*, C3b; *D*, C3c; *E*, C3d. The zinc concentrations were 2 μm (*black line*), 6 μm (*black dashed line*), 20 μm (*black dotted line*), 60 μm (*dot and dash line*), all shown in *black*, and 120 μm (*gray line*), 200 μm (*grey dashed line*), 600 μm (*gray dotted line*), all shown in *gray. Panels A–D* are drawn to the same scale for clarity of comparison. The zinc concentrations in micromolar are denoted numerically.

For C3 in 2 μm zinc, *M* was 5.0 nm, and *L* was 16 nm (Fig. 6*A*). These values were identical with those for C3 without zinc (43). For 6–60 μm zinc, *M* increased from 5.0 to 6.7 nm, *L* increased from 16 to 19 nm, and the area under the *P(r*) curve doubled. The changes indicated small amounts of C3 aggregate formation. For 120–600 μm zinc, the area under the *P(r*) curves underwent large increases together with increases in *L* from 16 to 50 nm. It was concluded that aggregates of C3-zinc had formed that were more than three times larger in size than the C3 monomer.

For C3u in 2 μm zinc, *M* was 5.1 nm, and *L* was 16 nm (Fig. 6*B*). These values agreed closely with those of C3u without zinc (43). Up to 60 μm zinc, the *P(r*) curves remained similar with modest increases in intensity and *L*. In 120–600 μm zinc, the *P(r*) intensity for C3u below 16 nm decreased, whereas the *P(r*) curves became significantly broader with *L* values that increased to about 50 nm. The *L* values for the C3u-zinc aggregates were similar to that for C3-zinc. For 120 and 200 μm zinc, the reduced *P(r*) intensities compared with those for C3 suggested that C3u aggregated less than C3.

For C3b in 2 μm zinc, *M* was 4.8 nm, and *L* was 16 nm (Fig. 6*C*), these being similar to those for C3 and C3u above. The *P(r*) curves remained almost unchanged with increases up to 60 μm zinc. Between 120 and 600 μm zinc, the *P(r*) curves changed in a similar manner to that of C3u, except that the intensity changes were smaller compared with those of C3 and C3u, suggesting that C3b aggregated less than C3u.

For C3c in 2 μm zinc, *M* was 4.5 nm, and *L* was 14 nm (Fig. 6*D*). Unlike C3, C3u, and C3b, the *P(r*) curve remained almost unchanged, with only a small increase in *L* to 18 nm when zinc concentration increased to 200 μm. In 600 μm zinc, M became 5.5 nm, and L became 22 nm together with slight increases in the *P(r*) intensity. These small changes reflected little C3c aggregation between 2 and 600 μm zinc.

For C3d in 2 μm zinc, *M* was 2.63 nm, and *L* was 6 nm (Fig. 6*E*; not drawn to scale). These values agreed with those of C3d without zinc (59). Between 2 and 600 μm zinc, the *M* and *L* values and the shape of the *P(r*) curves were almost unchanged, with only slight decreases in the *P(r*) intensity above 120 μm zinc that may result from slight C3d precipitation. These small changes also reflected little C3d aggregation between 2 and 600 μm zinc.

##### Scattering Curves of the FH-C3b-Zinc Complex

The FH-C3b complex in the presence of zinc was studied by x-ray scattering. The Guinier fits (not shown) were performed in the same *Q* ranges of 0.14–0.22 nm^−1^ for the apparent *R_G_* and *I*(*0*)/*c* values and 0.32 to 0.45 nm^−1^ for the *I*(*0*)/*c* base line. Each of C3b, FH and their 1:1 mixture was titrated with 2–600 μm zinc (Fig. 5, *C* and *D*). On the addition of zinc, both Guinier parameters for FH increased rapidly in agreement with those previously seen with FH-zinc (31, 32). Those for C3b increased more slowly, in agreement with Fig. 5, *A* and *B*. Those for the FH-C3b mixture increased with zinc to follow initially the results with FH-zinc. At a zinc concentration >100 μm, when C3b started to bind to zinc, both the *R_G_* and *I*(*0*)/*c* parameters decreased. These decreases above 100 μm zinc are attributable to the formation of very large complexes of FH and C3b with zinc, which precipitate out of solution and no longer contribute to the scattering curves. These results are consistent with ultracentrifugation that showed that the C3b-FH-zinc complexes precipitated (Fig. 3, *C* and *D*).

##### Alternative Pathway Activation Assay

**FIGURE 7.**
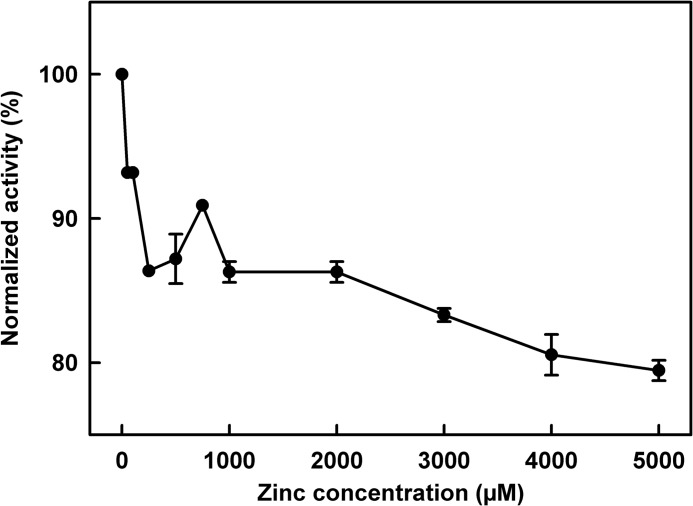
**Effect of zinc on alternative pathway activation in human serum.** Human serum was made 0, 100, 250, 500, 750, 1000, 2000, 3000, 4000, and 5000 μm in zinc. Radial diffusion activity assays were performed and normalized to 0 μm. The experiment was performed in triplicate, and the means ± S.E. of each measurement are shown.

To investigate whether zinc affected the complement alternative pathway, an alternative pathway hemolytic complement kit (“Experimental Procedures”) was used to study the effect of zinc sulfate on complement activation in human serum. Between 0 and 100 μm zinc, the activity decreased by ∼10% and between 100 and 5000 μm zinc the activity diminished by a further 10% (Fig. 7). The results are explained by the precipitation of C3b-FH-zinc complexes, thus reducing the availability of C3b to mediate its normal inflammatory response.

##### Computational Zinc Binding Predictions for C3, C3u, C3b, C3c, and C3d

Zinc binding sites are generally formed from four residues that are coordinated by zinc (35). To clarify the molecular basis of C3 self-association with zinc, surface zinc binding sites were predicted from seven crystal and solution structures for C3, C3u, C3b, C3c, and C3d (Table 1). These were submitted to the METSITE prediction server (37). METSITE was developed using relative residue positions and does not require side-chain atoms to be present; therefore, its predictions are applicable to low resolution structures. METSITE has an estimated site sensitivity of 77% and site selectivity of 44% for zinc predictions (see Table 3 of Ref. 37). Multiple partial zinc binding sites composed of His, Glu, and Asp residues were predicted on the surface of all five structures when the neural network output residue score was >0.7 to correspond to strong hits (Table 1). No well defined zinc binding sites were observed, and the number of sites varied from protein to protein. If the zinc binding capacity corresponds to the number of predicted zinc binding residues per 100 residues, C3 has 2.4 unique zinc binding site residues followed by C3b at 1.8–2.9 residues, C3u at 1.8 residues, C3c at 1.5 residues, and C3d with 1.0–1.3 residues. A separate prediction for the CUB domain (which is not present in C3c or C3d) showed no zinc binding residues in CUB from C3, and one His residue in CUB from C3u. Overall, the predictions show that weak zinc binding to the surfaces of C3, C3u, and C3b explains their oligomer formation. The comparative Table 1 predictions were consistent with the experimental ultracentrifugation and scattering data showing strong oligomerization in C3, C3u, and C3b and weaker effects on C3c and C3d.

**TABLE 1:** **METSITE predictions of zinc binding residues for the protein structures of C3, C3u, C3b, C3c, and C3d** The single neural network cutoff threshold was set at 0.7 in order to represent the log of the likelihood ratio scores of approximately 1.

Name	PDB code	Number of C3 domains	Number of residues in the PDB file	Unique zinc binding residues predicted METSITE >0.7	Number of unique zinc binding residues per 100 residues
C3	2A73	13	1611	38	2.4
C3b	2ICF	12	1545	45	2.9
C3b	2I07	12	1531	27	1.8
C3u	3MMQ	13	1531	28	1.8
C3c	2A74	10	1109	17	1.5
C3d	1C3D	1	294	3	1.0
C3d	1GHQ	1	307	4	1.3

**FIGURE 8.**
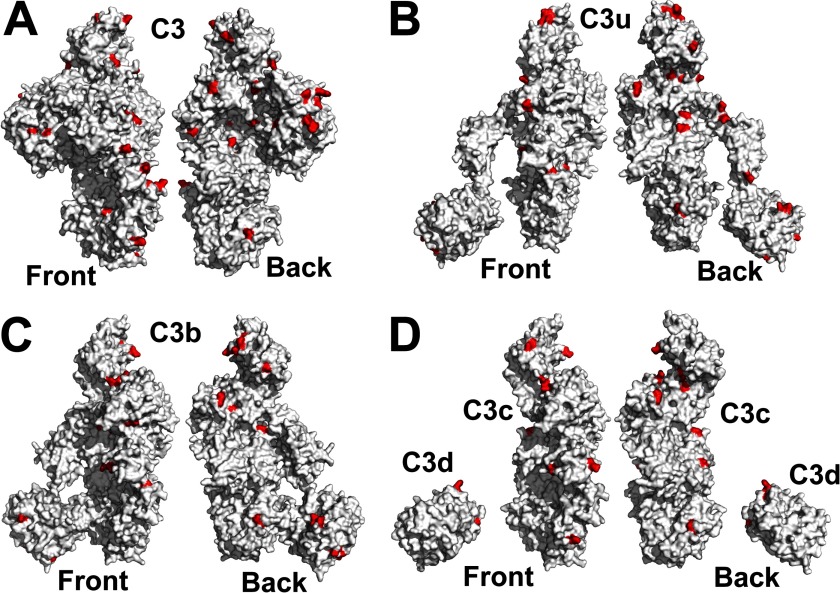
**Surface view of the METSITE predictions for C3, C3u, C3b, C3c, and C3d.** Each structure is displayed as the front view on the *left* and rotated 180° about the vertical axis to show the back view on the *right*. Residues highlighted in *red* represent the METSITE predictions that were registered above a neural network score of 0.7. The PDB codes are 2A73 for C3 (*A*), 3MMQ for C3u (*B*), 2I07 for C3b (*C*), 2A74 for C3c and 1C3D for C3d (*D*).

The front and back surfaces of the five C3 proteins showed that the partial zinc binding site predictions were broadly distributed (Fig. 8). Many sites were not reproducibly predicted between the five different structures; this variability is attributed to the weak zinc binding affinity. Given these moderate accuracies, zinc oligomer formation could not be assigned to specific residues in the five proteins. Nonetheless, the lack of zinc-induced oligomers for C3c and C3d is attributable to insufficient totals of weak zinc sites for daisy-chaining via zinc to form oligomers (Fig. 8*D*). When C3c and C3d are present together in C3, C3u, and C3b, the zinc sites in C3c and C3d were able to pair and cross-link C3, C3u, or C3b with zinc (Fig. 8, *A–C*). The large soluble C3-zinc oligomers, in contrast to the precipitation seen with C3u-zinc and C3b-zinc, are attributable to the different location of C3d relative to C3c in the three proteins.

To test whether METSITE was able to identify partial zinc binding sites, structures with zinc located at the interface between two distinct domains were tested (35). A nerve growth factor (PDB code 1SGF) was split into two domains (chains G and A) to predict the four ligands at the zinc binding site between them. In the separated domains, METSITE identified two clearly, namely His-202 and Glu-207 in domain G (scores of 0.91 and 0.98 respectively), plus a third one, Glu-51 (score of 0.55), but not the fourth His-58 in domain A. When the intact protein was analyzed, three of the four ligands were correctly predicted. A carbonic anhydrase (PDB code 1THJ) predicted one site (chain A His-117, score 0.8), but two were missed (chain B His-81 score 0.45, and chain B His-122, no score). This outcome was the same when the intact protein or the two separate domains were analyzed. The comparisons confirm that the METSITE predictions were moderately effective at detecting weak zinc binding sites.

## DISCUSSION

**FIGURE 9.**
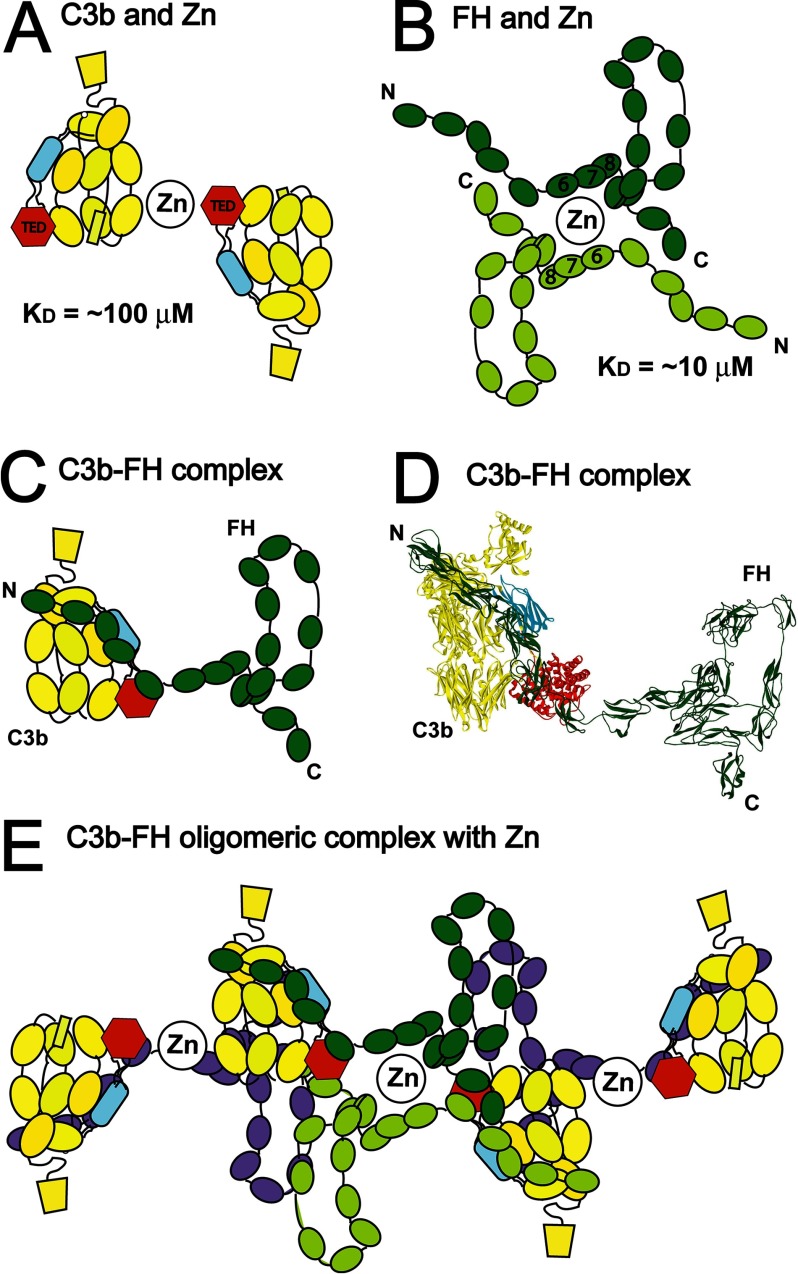
**Schematic outlines of the zinc-induced oligomerization of FH-zinc, C3b-zinc, and C3b-FH-zinc complexes.** *A*, a putative C3b dimer is shown in which the C3c and C3d regions in *yellow* and *red*, respectively in two monomers are cross-linked by zinc. Other cross-linked forms of C3b can also be formed by the daisy-chaining of the cross-linking. *B*, a putative FH dimer is shown in which the SCR-6/8 domains are cross-linked by zinc. These structures can also be daisy chained to form larger oligomers. *C* and *D*, the crystal structure of C3b with SCR-1/4 of FH is shown as a schematics form and as a molecular structure (PDB code 2WII) to which SCR-5/20 from the folded-back solution structure of FH SCR-1/20 (PDB code 3GAV; R. Nan, unpublished modeling) is added to indicate their interaction. C3b is shown in *yellow* (with the CUB domain in *blue* and the TED domain in *red*) and FH is shown in *green. E*, the formation of large aggregates of C3b-FH-zinc is shown. The daisy-chaining of the two dimers shown in *A* and *B* is accompanied by the formation of the C3b-FH complex to form an aggregated particle. Further FH monomers (in *purple*) are able to bind to the two C3b dimers, and these purple FH monomers are also able to bind zinc and dimerize further.

Our detailed study of the C3, C3u, and C3b interactions with zinc revealed that these proteins underwent different forms of self-association at zinc concentrations above 100 μm (Table 2). With zinc, C3 formed large oligomers, C3u and C3b precipitated, and C3c and C3d showed little self-association. Most importantly, strong precipitation was observed when zinc was added to the key physiological C3b-FH complex (Fig. 9, *C* and *D*), and the activation of the complement alternative pathway was detectably inhibited by zinc. These results suggest that in inflammatory conditions the pathophysiological release of zinc at unexpectedly high concentrations into the extracellular space has the capacity to perturb the complement system and initiate protein oligomer deposition.

**TABLE 2:** **Summary of the effect of zinc on the five forms of C3, FH and the C3b-FH complex** Aggregation is considered to be the sum of oligomer formation and precipitation. The outcomes of Fig. 1–6 are summarized using the following codes: A, aggregation; ma, minor aggregation; O, oligomer; P, precipitation; mp, minor precipitation; MP, major precipitation; NA, not applicable.

Protein	Fig. 1	Fig. 2	Fig. 3	Fig. 4	Fig. 5	Fig. 6	Overall
C3	O	O	NA	A	A	O	O
C3u	P	P	NA	A, P	A	O, P	P
C3b	P	P	NA	A, P	A	O, P	P
C3c			NA	ma	A		mp
C3d		mp	NA	ma	ma		mp
FH	NA	NA	O	NA	A	NA	O
C3b.FH	NA	NA	MP	NA	MP	NA	MP

Extracellular metal-induced protein aggregation is significant in several degenerative diseases such as in Alzheimer disease, amyloid and prion diseases, and age-related macular degeneration where extracellular zinc concentrations can reach high micromolar or even millimolar levels due to the release of zinc from neighboring cells (60, 61). The local elevation in extracellular zinc levels is likely to be the result of pathological events even though during neuronal activity, zinc is released into the synaptic cleft and is believed to reach local concentrations of 300 μm (62). A number of ratiometric zinc sensors estimate the intracellular bioavailable zinc levels to be in the nanomolar to picomolar range in resting cultured cells, which can be compared with the total intracellular zinc concentration of ∼100 μm (63). Although the extracellular bioavailable zinc levels are yet to be precisely determined, indications are that these will also be in the nanomolar to picomolar range, similar to intracellular bioavailable zinc levels. Thus bioavailable zinc levels in plasma are in a range of 20–210 pm (64). In plasma, the total zinc level remains steady at 14.7 μm even after a daily diet supplement with 80 mg zinc in the AREDS trials (38). Most plasma zinc (84%) is bound to human serum albumin with a *K_D_* of 1 μm, and α2-macroglobulin binds 15% of plasma zinc (65). Compared with *K_D_* values of ∼100 μm for C3/C3u/C3b-zinc binding and 10 μm for FH-zinc binding, human serum albumin acts as a scavenger of bioavailable zinc, and accordingly there is little risk of C3/C3u/C3b or FH self-association with zinc in normal plasma conditions. The same is likely to be true for the extracellular space in a resting stage. However, pathological events may trigger the release of high concentrations of zinc locally, especially in tissues where zinc concentrations are exceptionally high such as the RPE (25).

Zinc appears to be essential for the normal function of the retina, but its exact involvement in normal and pathological functions is unclear (27). Intracellular zinc is mostly bound to proteins including metallothioneins, carbonic anhydrase, and other zinc binding proteins (25, 27, 66). RPE cells are able to accumulate zinc after oral supplementation and retain this zinc for longer than any other tissues in the body, suggesting a special process for zinc storage and distribution in these cells (66). Zinc levels can also decrease in RPE cells under pathological conditions such as that seen in AMD (66), releasing zinc into Bruch's membrane where it could bind to proteins and induce their aggregation *in vivo*. These aggregated proteins then become part of sRPEds (28). We have shown that Bruch's membrane contains bioavailable zinc, especially in samples that contain substantial sRPEds, evidenced by their labeling in the presence of selective fluorescence sensors that only bind to bioavailable zinc (67). This bioavailable zinc could lead to the formation of the C3-zinc, C3u-zinc and C3b-zinc and C3b-FH-zinc complexes (Fig. 8*A*) as well as to those for FH-zinc complexes (31). Our zinc concentrations from 100 μm upwards, at which we observed oligomerization separately with each of C3, C3u, C3b, and with the C3b-FH complex, therefore have biomedical significance in the eye.

The zinc binding studies with C3/C3u/C3b show that these proteins exhibit different modes for their self-association in the presence of >100 μm zinc (Figs. 2, 5, and 6). Zinc has, therefore, been useful as a probe of structural differences between these three proteins, adding to the knowledge already obtained with the crystallography of C3 and C3b and the constrained scattering modeling of C3u (4, 43, 51). The more compact C3d-C3c arrangement in C3 promotes larger soluble oligomers with zinc, whereas the more extended C3d-C3c arrangements in C3u and C3b promote greater precipitation with zinc (Fig. 9*A*). The fact that zinc had little effect on each of C3c or C3d suggests that both of these are involved in zinc binding only when they are together in C3, C3b, or C3u. At zinc above 10 μm (30, 31, 32), strong oligomerization occurred for both the wild-type Tyr-402 and disease-related His-402 allotypes at the SCR-6/8 domains (32). Indefinite daisy-chains of cross-linked SCR-6/8 domains accounted for the large FH oligomers, starting from a simple dimer (Fig. 9*B*). Our new results showing that C3b self-associated with zinc and that the C3b-FH complex precipitated with 100 μm zinc have changed this understanding. The combination of separate C3b-zinc and FH-zinc self-associations in the C3b-FH complex promotes even greater amounts of oligomer formation (Fig. 9*E*). Each FH dimer will bind to two C3b dimers; in turn, each C3b dimer will bind to two FH dimers. Two separate weak zinc binding events with micromolar affinities becomes a much stronger interaction when both events occur simultaneously in different parts of the same complex (68).

FH is a major complement regulator and is expressed and secreted by many different cell types including the RPE (69). Factor H and C3 have been detected in retinal and RPE/choroidal tissues (70). The major physiological ligands of FH include C3b and its C3d fragment, heparan sulfate and other glycosaminoglycans, and C-reactive protein (71). All these ligands bind weakly to FH with micromolar affinities, as expected given the micromolar abundance of FH in serum. Thus C3b and C3d bind to FH with *K_D_* values of 0.6–1.6 and 2.6 μm, respectively (9, 72), heparin binds to two sites on FH with *K_D_* values of 1–3 μm (73), and C-reactive protein binds with a *K_D_* value of 4–15 μm (74). If C3b at 1 mg/ml is mixed with FH at 0.8 mg/ml, only 60–70% of the complex is formed with a *K_D_* value of 0.6–1.6 μm. This illustrates how complement regulatory control is achieved as the result of incomplete complex formation between its major ligands. When pathophysiological amounts of >100 μm zinc are present together with high levels of localized inflammation (when much C3b is formed), our results show that zinc will precipitate and remove the C3b-FH complex and free C3b. The normal mechanisms of complement control that involves multiple interactions with C3b, heparan sulfate, and C-reactive protein become perturbed (71).

In summary, we propose that during AMD-associated inflammation, two influencing factors need to be considered: (i) the release of 10–100 μm bioavailable zinc will affect the oligomerization and activity of FH (31); (ii) if zinc concentrations increase even further, C3b will also form oligomers, but more importantly any C3b-FH complexes that are formed in Bruch's membrane will precipitate in >100 μm zinc. The C3b-FH-zinc precipitates will contribute to sRPEd formation. This may explain the presence of FH, C3b, and other complement proteins in sRPEds (11). However, although the reduction of FH levels will promote uncontrolled inflammation, the precipitation of C3b-zinc and C3b-FH-zinc complexes will limit that. Therefore, clinically, zinc might contribute to the early and late stages of AMD in two distinct ways (75). Whether the beneficial effects of zinc supplementation (38) is related to the precipitation of C3b-FH-zinc complexes will need to be determined.

## Footnotes

4 Our previous study (43) reported *s* values of 7.85 ± 0.05 S for C3 and 7.44 ± 0.07 S for C3u, when these should have been presented as *s*_20,_*_w_* values of 8.49 ± 0.03 S and 8.08 ± 0.02 S respectively.

## Footnotes

3 The abbreviations used are:

C3: complement component 3
FH: Factor H
AMD: age-related macular degeneration
SCR: short complement regulator
RPE: retinal pigment epithelium
sRPEd: sub-RPE deposit
S: siemens
ANA: anaphylatoxin domain
CUB: complement C1r/C1s-UEGF-BMP1 domain
TED: thioester-containing domain.
